# Heart Rate Variability and Physical Demands of In-Season Youth Elite Soccer Players

**DOI:** 10.3390/ijerph18041391

**Published:** 2021-02-03

**Authors:** Javier Sánchez-Sánchez, Javier Botella, Jose Luis Felipe Hernández, Manuel León, Víctor Paredes-Hernández, Enrique Colino, Leonor Gallardo, Jorge García-Unanue

**Affiliations:** 1 Faculty of Sport Sciences, Universidad Europea de Madrid, Calle Tajo, s/n, Villaviciosa de Odón, 28670 Madrid, Spain; javier.sanchez2@universidadeuropea.es (J.S.-S.); joseluis.felipe@universidadeuropea.es (J.L.F.H.); 2 Institute for Health and Sport (iHeS), Victoria University, Melbourne 3011, Australia; 3 Grupo IGOID, University of Castilla-La Mancha, Avda Carlos III, 45004 Toledo, Spain; manuel.leonjimenez@yahoo.es (M.L.); enrique.colino@uclm.es (E.C.); leonor.gallardo@uclm.es (L.G.); jorge.garciaunanue@uclm.es (J.G.-U.); 4 Universidad Camilo José Cela, Calle Castillo de Alarcón, 49, Villanueva de la Cañada, 28692 Madrid, Spain; vparedes@ucjc.edu

**Keywords:** HRV, physical performance, training load, youth, soccer

## Abstract

Monitoring fatigue and performance is important for adjusting training loads in soccer. Therefore, knowing the status of the player when applying a training stimulus is key to optimizing the players’ development. This study aims to evaluate the interaction between internal and external load, during training and matches, in an elite youth soccer team. Methods: seventeen youth players of the highest Spanish category were monitored with GPS devices during training and matches, as well as recording their nocturnal heart rate variability (HRV). We employed a linear mixed model to assess the physical demands between training and matches, and to compare the HRV variables. Results: a higher total distance (+2993.35–5746.56 m; ES = 1.4), distance at high intensity (+641.24–1907 m; ES = 1.5), sprint distance (+350.46–795.05 m; ES = 2.1), number of sprints (+18.38–41.58; ES = 1.9), and number of repeated sprints (+5.91–15.30; ES = 1.7) (all *p* < 0.001), but not in the number of accelerations, were reported during the matches when compared to the training sessions during the 11 weeks. The analysis of the HRV variables showed no significant differences between the accumulated values during a training week, providing similar results pre-match or post-match (*p* > 0.05). The LF/HF_RATIO_ showed a negative influence on the total distance ran, distance at high intensity, distance in sprint, number of sprints, and repeated sprint. RR_MEAN_ was positively related to the sprint number. Conclusion: the results of the present study suggest that nocturnal HRV variables are not different between pre-match and post-match. Furthermore, it suggests that LF/HF_RATIO_ and RR_MEAN_ during pre-match can determine the external load that the player will be able to complete during the match.

## 1. Introduction

GPS technology has helped team sports to better understand the physiological demands and characteristics of the game. External loads obtained in soccer have been related to the player position [1,2] and type of match [3]. A good management of the acute and chronic workload relationship has been shown to be able to predict the occurrence of injuries [4,5]. Therefore, external load monitoring during training and matches is essential for optimal workload programming for soccer players

Exercise performed by soccer players during their training and matches includes a wide variety of stimuli (i.e., sprinting, jumping, and kicking). These stimuli will have an impact on their physiological, neuromuscular, and psychological readiness [6]. Isolated analysis of external loads does not guarantee a correct distribution of the workload due to individual heterogeneity response and player adaptation [7]. Different monitoring tools allow for tracking physiological, neuromuscular, and psychological demands, and is now widely possible due to advances in technology [8]. Among these tools, questionnaires, blood samples, oxygen consumption, and heart rate monitoring [7] have proven to be valid tools for estimating the physiological internal load. However, these can be time consuming in the collection and interpretation of the results, and sometimes require advanced equipment. Therefore, the integration of multiple internal and external load systems will facilitate the evidence-based decisions for optimizing in-field performance.

Heart rate variability (HRV) is among the monitoring tools that are increasing in popularity due to its measurement ease and validity to provide an internal load measure [9,10]. Many authors suggest combining external and internal parameter for the correct evaluation of the training response. HRV allows for evaluation of different components of the sympathetic and parasympathetic nervous system [11]. The root mean square of successive differences (RMSSD) in R-R intervals is the most often used variable to assess parasympathetic level because it is less influenced by environmental and methodological variables [12]. Parasympathetic tone, through HRV, has also been shown to be associated with readiness to perform and to be a good monitoring tool to optimize the training process [10,13]. Previous studies in soccer have combined external load variables with HRV as an indicator of the internal load during preseason [14] and competitive periods [15]. Interestingly, Buchheit, Chivot [16] identified the RMSSD as a predictor of the changes in the aerobic capacity of players or the fluctuations in the distance traveled at high intensity in elite soccer players. However, whether the HRV variables would predict performance in training and matches in youth soccer players remains equivocal.

The relationship between HRV and the external load parameters during the matches could be an effective method to assess the player’s readiness for the game. However, there is no scientific evidence on the influence of internal load on physical performances during matches in youth soccer players. Therefore, the aim of this study was to evaluate the interaction between internal and external load, both during training and match games, and the concurrent changes in nocturnal HRV variables in an elite youth soccer team.

## 2. Methods

### 2.1. Sample

Seventeen soccer players of the highest Spanish junior category (18.8 ± 0.4 years, 176.0 ± 0.3 cm, 70.1 ± 6.7 kg) volunteered to take part in this study. Goalkeepers were excluded from the study due to their lower external load demands. The remaining players were informed of the use of the training and match data for the purpose of this study and accepted the continued monitoring of HRV through signed consent. The study was approved by the Clinical Research Ethics Committee of the Castilla-La Mancha Health Service (Ref: 489/24022020) and approved in accordance with the latest version of the Declaration of Helsinki.

### 2.2. Study Design

GPS monitors were used to track the external load demands during 11 weeks of training sessions and match games of the highest Spanish youth category. The duration of the training sessions were 90 min, involving mainly small-side game (SSG) tasks adapted to game conditions. SSG has been demonstrated as an effective method to increase the external load without affecting the internal load [17] and improving aerobic fitness [18].

The external load of both training sessions and matches were collected through the use of 15 Hz GPS devices (SPI Pro, GPS Sports^®^ Canberra, Australia). The GPS devices were provided to the players from the first day of weekly training (Wednesday) until the day of the game (Saturday or Sunday). On Mondays, players completed recovery sessions and Tuesdays were established as a rest day. Each session had to collect at least 60 min of recording and the players had to finish the training session to be able to take it into account for analysis. Seven recordings did not meet these conditions and were not included in the analysis.

The variables used to record the external load were total distance (m), high-intensity distance (m > 19.1 km·h^−1^), total sprint distance (m > 25.1 km·h^−1^), number of sprints, sprint repetitions, and accelerations [1]. Repeated sprints are maximum or submaximal efforts of less than 10 s duration with a rest interval of less than 60 s between each sprint [19]. The acceleration threshold was described by Johnston, Watsford [20] determining that an acceleration must exceed 2.75 m·s^−2^ and surpass a duration of 0.5 s.

Measurement of HRV was performed using the Bodyguard 2 device (Firstbeat Technologies Ltd., Jyväskylä, Finland) during sleep, excluding the initial thirty minutes and analyzing the following 4 h to capture the slow wave sleep (SWS), which usually takes place during this period [21]. The HRV recording days were the same as the GPS recordings (from Wednesday to Sunday).

The data collected during each week were divided into three different periods (Table 1): (1) weekly accumulation (average measurements of the whole week without taking into account the day before the match), (2) pre-match (HRV recorded the night before the matches game), and (3) post-match (HRV recorded the night after the matches game). The variables selected were stress balance or ratio between stress and recovery reactions during the night [22]. The stress balance is an index that shows the difference between the 1 min segments during the sleeping period that were classified as stressful (high heart rate and low variability) or as recovery (heart rate is low and variability is high) calculated by the Firstbeat software (Firstbeat Technologies Ltd., Jyväskylä, Finland). The other parameters were low-frequency/high-frequency ratio (LF/HF_RATIO_) (proposed to be an indicator of the sympathovagal balance), the average time between two successive R-R intervals (RR_MEAN_), and the root mean square of successive differences (RMSSD) in R-R intervals [11].

### 2.3. Statistical Analysis

Data are presented in graphs and tables as mean ± SD. Data in the results are shown as the lowest to highest difference between the training and the match session, effect size, and *p* value. The training load of sessions is shown as an average of the three training days. The dataset presents an unbalanced repeated measured data due to the players’ difference in number of training and match games. Therefore, a linear mixed model was used to compare the physical demands between training and matches in the different weeks and to compare the HRV variables between the mean of the week, the night before the matches (pre-match), and the night after the matches (post-match), whereas any interactions were identified using Bonferroni post hoc pairwise comparisons. The significance level was set at *p* < 0.05. In addition, confidence interval (CI of 95%) and the effect size (ES; Cohen’s d) were calculated to identify the magnitude of change. ES was evaluated by the following criteria: trivial, 0–0.2; small, 0.2–0.5; moderate, 0.5–0.8; and large, >0.8. Finally, several linear regressions were estimated using the pre-match heart rate variability parameters as independent variables and the results from physical demands during matches as dependent variables. The variance inflation factor (VIF) was calculated to conclude the nonexistence of multicollinearity in the model. Data were analyzed using the statistic software SPSS V21.0 (SPSS Inc., Chicago, IL, USA).

## 3. Results

**Figure 1:**
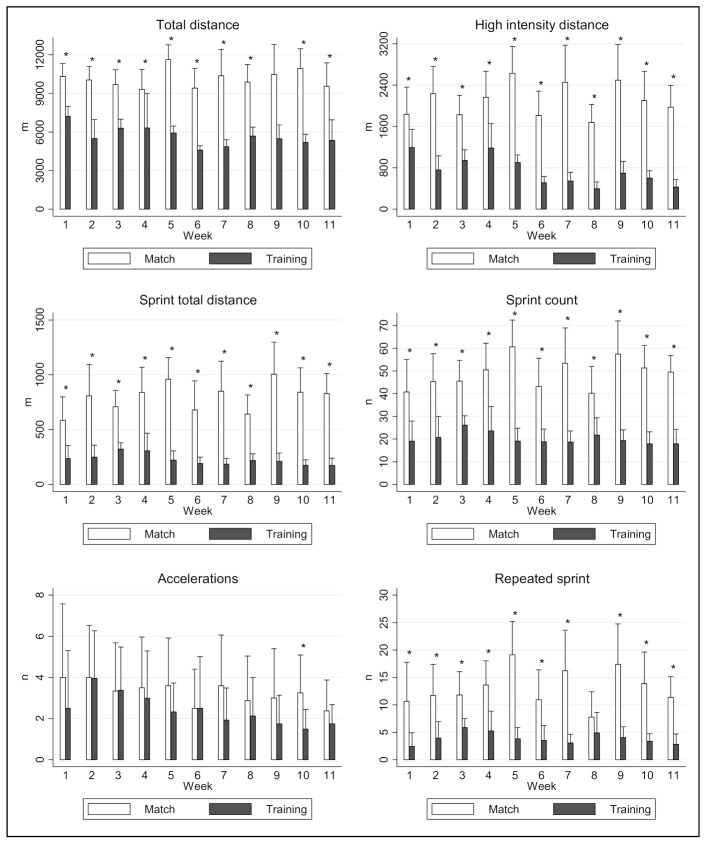
Physical demands of training and match play in young elite soccer players. * Differences between training sessions and matches (*p* < 0.05). Bars display mean ± standard deviation.

The results obtained during the matches, analyzed for the youth elite soccer players, revealed greater total distances traveled (+2993.35–5746.56 m; ES = 1.4; *p* < 0.001), high-intensity distances (+641.24–1907 m; ES = 1.5; *p* < 0.001), total sprint distance (+350.46–795.05 m; ES = 2.1; *p* < 0.001), sprint count (+18.38–41.58; ES = 1.9; *p* < 0.001), and repeated sprints (+5.91–15.30; ES = 1.7; *p* < 0.001) compared to the results obtained during training sessions. However, the number of high-intensity accelerations (>2.75 m·s^−2^) was similar during games and training (*p* > 0.05, Figure 1).

On the other hand, the analysis of the HRV variables showed no significant differences between the accumulated value of training weeks or the results obtained pre-match and post-match (*p* > 0.05, Table 1).

The regression analysis between pre-match nocturnal HRV and in-match external load revealed a negative influence of the LF/HF_RATIO_ on the total distance traveled, distance at high intensity, distance in sprint, number of sprints, and repeated sprint (Table 2; *p* < 0.05). The RR_MEAN_ had a significant and positive relationship with the sprint count (*p* < 0.05). On the other hand, although at an individual level the RR_MEAN_ interval showed a direct relationship on accelerations (*p* < 0.05), the regression was not significant.

## 4. Discussion

The main findings of this study show that, in youth soccer players, the external loads monitored between training and match games were different for multiple speed and sprint variables. It also suggests that nocturnal HRV parameters before or after matches are not different or sensitive to fatigue status. Furthermore, pre-match nocturnal LF/HF_RATIO_ and RR_MEAN_ significantly correlated with multiple external load variables recorded during the match.

The present study showed that external loads recorded during training and competitive soccer matches were different in total distance, distance at high speed, distance in sprint, number of sprints, and repeated sprints, but not in the number of accelerations in youth soccer players. These results are in accordance with previous studies of professional elite youth soccer players [23,24], and close to professional players [2,25] for both total and high-intensity distance. These results were also reported in previously studies by Henderson, Cook [26] and Rebelo, Brito [27], where it was shown that the physical demands of a match are above those experienced during training. In this sense, Stevens [25] suggested that the number of accelerations and decelerations performed in training are similar to those seen in a match, despite having a different total running distance. This may be partly due to the use of SSG in training, increasing the physical load through the use of accelerations [28]. Given these results, we suggest that external load should be monitored during both training and match games to accurately reflect the players’ external load.

There are currently no established external load standards in youth soccer due to the large differences in their capacity to train, as well as their individual biological growth [17]. Thus, the measurement of HRV, as a noninvasive tool, has been suggested to be a good option to control the internal load of players, especially those who are in growth period. In addition, the development of new mobile devices means that tests performed under laboratory conditions can be reproduced in the field, with a lower cost and thus facilitating the control of HRV [29]. Our results showed that the nocturnal HRV was not significantly different between the values accumulated during the week, and to those collected before or after the game. These results coincide with the study by Oliveira, Leicht [30] who showed that changes in the HRV during the season are typically low. This is due to the fact that during the season there are no large fluctuations in the training load [31], and only high stressors may be capable of significantly modifying these HRV variables. Therefore, the external load must be quantified to better understand the effects of this internal–external load relationship on the athlete’s performance [6]. The present findings indicate that internal load control in youth soccer players, by means of nocturnal HRV, was not affected by the timing of measurement (before or after match). Therefore, our results suggest that monitoring the internal load, by means of nocturnal HRV, is not influenced by the day of the week when the measurements were taken. Whether this reflects a lower sensitivity of nocturnal HRV to detect changes in fatigue status, or simply unchanged status, remains to be fully established in future research.

Our study shows that HRV parameters were related to in-match performance indicators. The pre-match cardiac variable most sensitive to high-intensity actions was the LF/HF_RATIO_, followed by RR_MEAN_. Our findings agree with those described by Bricout, DeChenaud [32] and Edmonds, Sinclair [33], showing that players who show a parasympathetic reduction (through nocturnal HRV variables) during the night have worse performance and a slower recovery. This is in line with our findings, where a higher LF/HF_RATIO_ before the game (suggesting a lower parasympathetic activation) was linked to a lower performance in the game. Our results are consistent with the results of Cornforth, Campbell [34] who proposed that by measuring the HRV during the morning before the game, it would be possible to make adjustments and have a greater control of the homeostatic equilibrium of the player. The results of this study show that HRV variables may be a good indicator of readiness to perform and match performance (sprint and speed variables) in youth soccer players. Whether our results would have been similar if HRV measures had been obtained during the morning remains to be elucidated.

At elite level where loads are high and performance gains are small, accurately monitoring internal and external load would allow for better prescription of the optimal training, and potentially give a competitive advantage. Measuring individual changes in fitness and fatigue markers allows the quantification of individual response and adaptation to training. This information would allow coaches to better understand the impact of the programmed loads on players, discovering tolerance thresholds. One of the limitations of the present study was that only the second half of the season was monitored and therefore no external or internal load records during the first part of the season were available. Furthermore, these results should be further explored with HRV variables obtained in different settings (e.g., morning assessment).

## 5. Conclusions

The average external load of training sessions is lower than that produced during matches in multiple sprint and speed variables. Furthermore, the weekly monitoring of HRV before or after a game is not sensitive to discriminate fatigue status and remains stable. Lastly, nocturnal pre-match HRV indices are related to the external load that the player will perform during the game. The continuous monitoring of the HRV may allow for optimization of the training load prescription in elite young soccer players.

## Figures and Tables

**Table 1:** Automatic cardiac responses during training nights, pre-match and post-match. Values are shown as mean ± standard deviation.

	Accumulated	Pre-Match	Post-Match	*p*
Stress Balance	0.66 ± 0.37	0.64 ± 0.45	0.70 ± 0.32	0.78
LF/HF_RATIO_	1.49 ± 0.57	1.52 ± 0.54	1.60 ± 0.72	0.52
RR_MEAN_	1189.89 ± 223.42	1248.89 ± 131.66	1248.68 ± 112.41	0.17
HR	50.42 ± 9.47	48.04 ± 5.06	48.05 ± 4.33	0.17
RMSSD	68.89 ± 28.89	73.38 ± 31.28	75.22 ± 29.93	0.93

LF = low frequency; HF = high frequency; RR_MEAN_ = average time between two successive R-R intervals; HR = heart rate; RMSSD = root mean square of successive differences of consecutive R-R intervals.

**Table 2:** Regression analysis of the influence that automatic cardiac responses have during physical demands in young elite soccer players.

	Distance	High-Intensity Distance	Sprint Count	Accelerations	Repeated Sprint	Sprint Distance
Stress balance	−152.86 ± 759.37	101.94 ± 272.01	−5.72 ± 5.19	1.00 ± 1.31	−1.29 ± 2.83	10.76 ± 103.31
LF/HF_RATIO_	−1230.84 ± 544.40 *	−504.50 ± 195.015 *	−11.87 ± 3.72 *	−0.30 ± 0.94	−4.36 ± 2.03 *	−229.47 ± 74.07 *
RR_MEAN_	5.04 ± 2.49	1.52 ± 0.89	0.036 ± 0.017 *	−0.00 ± 0.00 *	0.08 ± 0.01	0.53 ± 0.34
RMSSD	1.43 ± 11.25	−4.82 ± 4.03	−0.094 ± 0.08	−0.00 ± 0.02	−0.03 ± 0.04	−1.60 ± 1.53
Constant	6630.35 ± 3270.28	1692.02 ± 1171.46	37.85 ± 22.37	15.78 ± 5.65 *	4.55 ± 12.22	690.74 ± 444.94
R^2^	0.40 *	0.40 *	0.46 *	0.271	0.31	0.46 *

LF = low frequency; HF = high frequency; RR_MEAN_ = average time between two successive R-R intervals; HR = heart rate; RMSSD = root mean square of successive differences of consecutive R-R intervals. Values shown are mean ± standard deviation; * indicates *p* < 0.05.

## Data Availability

The data that support the findings of this study are available from the corresponding author, upon reasonable request.

## Abbreviations

HRV	Heart Rate Variability
RMSSD	Root Mean Square of Successive Differences in R-R intervals
SSG	Small-Side Games
